# Th17/Treg imbalance in inflammatory bowel disease: immunological mechanisms and microbiota-driven regulation

**DOI:** 10.3389/fimmu.2025.1651063

**Published:** 2025-10-08

**Authors:** Yuyuan Hu, Yuhang Yang, Yan Li, Qiang Zhang, Wei Zhang, Jinghan Jia, Zhuoyi Han, Jinxi Wang

**Affiliations:** ^1^ Division of Colorectal Surgery, Shanxi Bethune Hospital, Shanxi Academy of Medical Sciences, Third Hospital of Shanxi Medical University, Tongji Shanxi Hospital, Taiyuan, China; ^2^ Neurology, Shanxi Bethune Hospital, Shanxi Academy of Medical Sciences, Third Hospital of Shanxi Medical University, Tongji Shanxi Hospital, Taiyuan, China

**Keywords:** inflammatory bowel disease, intestinal immune response, Th17 cells, Treg cells, gut microbiota, microbiota imbalance

## Abstract

Inflammatory bowel disease (IBD) is a group of conditions characterized by chronic and recurrent intestinal inflammation, primarily including Crohn’s disease (CD) and ulcerative colitis (UC). The pathogenesis of IBD is closely linked to abnormal immune responses, particularly T-cell mediated immune reactions. Th17 cells promote persistent intestinal inflammation by secreting pro-inflammatory cytokines such as IL-17, while regulatory T (Treg) cells help maintain immune homeostasis by secreting anti-inflammatory cytokines like IL-10 and TGF-β. In patients with IBD, Th17 cell function is enhanced, whereas Treg cell function is impaired or their numbers are reduced, leading to an imbalance in the immune system and exacerbating intestinal inflammation. The gut microbiota plays a crucial role in the immune regulation of IBD. Dysbiosis can lead to excessive activation of Th17 cells and suppression of Treg cell function, further aggravating clinical symptoms. Studies have shown that restoring gut microbiota balance through probiotics, antibiotics, dietary interventions, or fecal microbiota transplantation can not only improve immune responses but also restore the balance between Th17 and Treg cells, which has a positive impact on IBD treatment. This review summarizes how gut microbiota modulates the Th17/Treg cell balance to influence IBD immune responses and explores therapeutic strategies targeting Th17/Treg balance, including cytokine antagonists and immunosuppressive agents, which provide new directions and approaches for clinical IBD treatment.

## 1 Introduction

Inflammatory bowel disease (IBD) encompasses a group of chronic and recurrent inflammatory disorders affecting the intestines (1). It arises from the abnormal activation of the intestinal immune system, resulting in persistent intestinal inflammation that may endure for years or even a lifetime (2). Although the precise etiology of IBD remains elusive, factors such as genetic susceptibility, immune dysregulation, and environmental influences significantly contribute to its pathogenesis (3). The clinical characteristics of IBD predominantly involve chronic inflammation of the intestinal mucosa and dysregulation of the immune system (4). Despite variations in clinical manifestations, affected sites, and complications, all these aspects reflect the detrimental effects of immune system dysfunction on gut health.

The role of the immune system in inflammatory bowel disease (IBD) has garnered increasing attention, particularly regarding the central role of T cells in the immune response, specifically Th17 and Treg cells (5). In a healthy intestinal immune system, a dynamic equilibrium between Treg and Th17 cells is maintained (6). However, studies indicate that in patients with IBD, excessive activation of Th17 cells and insufficient function of Treg cells often disrupt this balance (5). Th17 cells, characterized by the secretion of IL-17, exhibit significantly elevated IL-17 levels in the intestinal mucosa and serum of active IBD patients (6). This suggests that IL-17, secreted by activated T cells, plays a pivotal role in the induction and maintenance of mucosal inflammatory responses in IBD. Treg cells, a subset of T cells, exert anti-inflammatory effects and sustain immune homeostasis by secreting anti-inflammatory cytokines such as IL-10 and TGF-β (6).

Recent studies have demonstrated that the gut microbiota plays a crucial role in the immune regulation of inflammatory bowel disease (IBD) (7). The diversity of the gut microbiota and its metabolic products not only influence the integrity of the intestinal barrier but also regulate the nature and intensity of immune responses by modulating the balance between Th17 and Treg cells (8). Dysbiosis may lead to excessive activation of Th17 cells and suppression of Treg cell function, thereby exacerbating the immune response in IBD (9). Consequently, restoring the balance of the gut microbiota has emerged as a promising strategy for the treatment of IBD (10). Current research indicates that probiotics, antibiotics, dietary interventions, and fecal microbiota transplantation can modulate the gut microbiota, improve the Th17/Treg cell balance, and positively influence the treatment and prognosis of IBD (11, 12). This review aims to explore the role of the gut microbiota in regulating the balance between Th17 and Treg cells in the onset, progression, and immune regulation of IBD (13). We will analyze the immune mechanisms underlying the imbalance of Th17/Treg cells and elucidate the regulatory role of the gut microbiota in this process. By delving into the mechanisms by which the gut microbiota regulates immune responses, this article aims to provide new theoretical insights and approaches for the early diagnosis, treatment, and development of immune modulation strategies for IBD. Furthermore, we will review the current progress in research on modulating the Th17/Treg balance and discuss the future prospects of gut microbiota modulation in the immune treatment of IBD.

## 2 The role of Th17 and Treg cells in inflammatory bowel disease

### 2.1 Mechanisms associated with the pathogenesis of inflammatory bowel disease

In the pathogenesis of inflammatory bowel disease (IBD), multiple key pathways exhibit heterogeneous effects on the activation of distinct cell types, with various combinations of these cells potentially playing a crucial role in the manifestation and progression of the disease (14). Genetic loci associated with IBD can be broadly categorized into several major pathways, including innate immune responses, intestinal barrier function, microbial defense, reactive oxygen species (ROS) production, and antimicrobial activity (15). Recent studies have demonstrated that certain receptors related to homing and migration, such as CD62L, C-C chemokine receptor CCR7, αEβ7 integrin, α4β7 integrin, IL-23R, IL-17R, CCR4, CCR5, and CCR9, are pivotal in the pathogenesis of IBD (16, 17). Notably, the expression of these receptors in Treg and Th17 cells is essential for maintaining intestinal immune homeostasis (18, 19).

Th17 and Treg cells are integral to the pathogenesis of inflammatory bowel disease (IBD). Th17 cells promote local inflammatory responses by secreting cytokines, including IL-17 and IL-22, which enhance the immune responses of intestinal epithelial cells and contribute to intestinal immune imbalance (20). Conversely, Treg cells suppress excessive immune responses and uphold immune tolerance in the gut by secreting anti-inflammatory cytokines, such as TGF-β and IL-10 (21). Research has indicated that Treg cell dysfunction in IBD patients, often stemming from abnormal expression of homing receptors, hinders their ability to migrate effectively to the gut, thereby compromising their regulatory role in intestinal immune responses (22). This imbalance leads to excessive activation of inflammatory responses, ultimately contributing to the onset and progression of IBD (23). Consequently, the interaction between Th17 and Treg cells, along with the balance of their roles in intestinal immune regulation, is central to understanding the pathogenesis of IBD (24).

### 2.2 The role of Th17 cells

#### 2.2.1 Differentiation and function of Th17 cells

Th17 cells are a subset of pro-inflammatory T cells characterized by the expression of interleukin-17 (IL-17) and retinoic acid receptor-related orphan receptor γt (25). In patients with inflammatory bowel disease (IBD), serum levels of cytokines that promote the differentiation of naïve CD4+ T cells into Th17 cells are significantly elevated (26). Upon stimulation with specific cytokines, Janus kinase 2 (JAK2) phosphorylates STAT3 in naïve CD4+ T cells (27). As a key transcription factor, phosphorylated STAT3 (p-STAT3) enhances the expression of downstream target genes such as RORC and IL17A, thereby facilitating the differentiation of Th17 cells (28).

Gut microbiota promotes the differentiation of naïve CD4+ T cells into Th17 cells by inducing antigen-presenting cells (APCs) to secrete cytokines such as IL-6 and IL-23, or by directly influencing them through their metabolites (29). The retinoic acid receptor-related orphan receptor γt (RORγt) is a Th17 cell-specific transcription factor that directly regulates Th17 cell differentiation (30). Various transcription factors, including IRF4, BATF, HIF-1, JunB, IL-6, IL-22, TGF-β, IL-1β, and IL-23, enhance Th17 cell differentiation by upregulating RORγt expression, which is subsequently regulated by the STAT3 signaling pathway (31). TGF-β is crucial for Th17 cell differentiation, as it not only promotes RORγt expression but also inhibits IL-17 expression induced by RORγt (32). Additionally, TGF-β facilitates IL-22 production through Smad2 activation, which can be inhibited by TNF receptor-associated factor 6 (TRAF6) (33). IL-6, an essential molecule, enhances RORγt gene expression via the JAK2-STAT3 signaling pathway, thereby facilitating Th17 cell differentiation (30). IL-1β promotes Th17 polarization through the IL1R/PI3K-mTOR signaling pathway while simultaneously inhibiting TGF-β-induced Foxp3 expression in CD4+ T cells, thus suppressing Treg cell differentiation (34). Furthermore, IL-21, secreted by Th17 cells, upregulates IL-17 and RORγt expression and promotes Th17 cell differentiation through the STAT3 signaling pathway (31).

In an inflammatory state, Th17 cells migrate from the bloodstream to the sites of inflammation within the gut, where they accumulate (35). This migration results in heightened activation of the gastrointestinal mucosa, triggering an intestinal immune response that increases the release of harmful cytokines and causes mucosal damage (36). Concurrently, the number of Treg cells increases to compensate for this damage. This process illustrates that during inflammation, Th17 cells infiltrate the affected areas and secrete the pro-inflammatory cytokine IL-17A (37).

#### 2.2.2 Pro-inflammatory role of Th17 cells in inflammatory bowel disease and its mechanisms

Th17 cells play a crucial role in the pathogenesis of inflammatory bowel disease (IBD) (36). Specifically, Th17 cells activate intestinal epithelial cells (IECs) and immune cells through the secretion of IL-17 and other cytokines, such as IL-6 and TNF-α, thereby triggering an inflammatory response (38). IL-17 exerts its pro-inflammatory effects through several mechanisms: either alone or in combination with tumor necrosis factor (TNF-α), IL-17 acts on IECs to promote the secretion of inflammatory mediators, chemokines, and proteases, including IL-6, IL-8, inducible nitric oxide synthase (iNOS), CXCL8, TNF-α, matrix metalloproteinase (MMP), and granulocyte-macrophage colony-stimulating factor (GM-CSF) (39). These factors induce inflammation and facilitate the recruitment, activation, and migration of neutrophils to target tissues, ultimately leading to mucosal damage (40).

**Figure 1:**
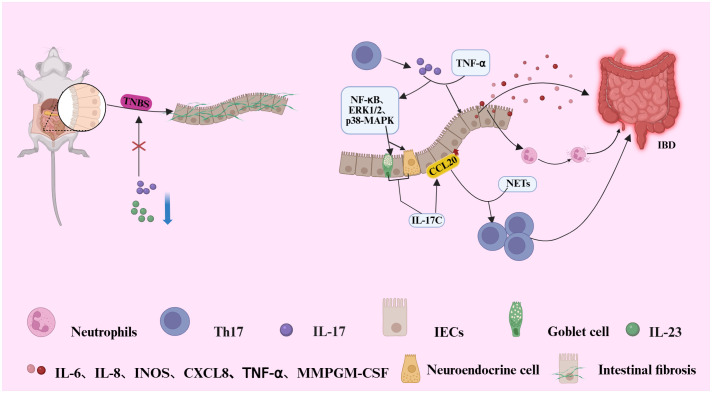
Illustrates the pathogenic mechanism by which IL-17 and TNF-α synergistically activate NF-dB, ERK1/2, and p38 MAPK signaling pathways in intestinal epithelial cells (IECs). This activation induces the secretion of IL-17C by neuroendocrine and goblet cells, subsequently upregulating the chemokine CCL20 in IECs. Elevated CCL20 expression, in conjunction with neutrophil-derived neutrophil extracellular traps (NETs), facilitates the recruitment and activation of Th17 cells, thereby amplifying the inflammatory response. Furthermore, the IL-17 produced by these recruited Th17 cells further amplifies inflammation through a direct effector mechanism.IL-17 (alone or with TNF-α) stimulates intestinal epithelial cells to secrete a suite of inflammatory factors (e.g., IL-6, IL-8, GM-CSF, MMPs). This promotes neutrophil recruitment and activation, leading to tissue inflammation and mucosal damage. In a murine model of colitis induced by 2,4,6-trinitrobenzene sulfonic acid (TNBS), inhibition of IL-23 or IL-17 signaling significantly attenuates intestinal fibrosis, highlighting a pivotal role for Th17 cells in fibro genic progression during IBD pathogenesis.

IL-17 collaborates with TNF-α to activate the NF-κB, ERK1/2, and p38 signaling pathways, which induce the secretion of IL-17C from intestinal neuroendocrine cells and goblet cells, thereby promoting the expression of the Th17 chemokine CCL20 in intestinal epithelial cells (IECs) (41). The upregulation of CCL20 expression, along with the formation of extracellular traps (NETs) by neutrophils, further enhances the recruitment and activation of Th17 cells, exacerbating the inflammatory response (42). In mouse models, the inhibition of IL-23 or IL-17 has been shown to significantly reduce 2,4,6-trinitrobenzenesulfonic acid (TNBS)-induced intestinal fibrosis, indicating that Th17 cells play a crucial role in the development of intestinal fibrosis (43). IL-17 induces excessive immune responses by modulating the intestinal immune environment, thus promoting the onset of fibrosis (43). It not only directly activates intestinal immune cells but also exacerbates tissue remodeling by inducing epithelial and stromal cells to secrete fibrosis-related factors, such as TGF-β and matrix metalloproteinases. Further studies have demonstrated that the inhibition of the IL-23/IL-17 signaling pathway can mitigate the progression of intestinal fibrosis, providing a novel therapeutic target for the treatment of inflammatory bowel disease (IBD) (see **Figure 1**) (42).

### 2.3 The role of Treg cells

#### 2.3.1 Treg cells maintain immune tolerance by secreting anti-inflammatory cytokines (e.g., IL-10, TGF-β)

In the pathogenesis of inflammatory bowel disease (IBD), the dysfunction of Treg cells not only impacts their survival but may also serve as a critical factor in the onset and progression of the disease (44). Under homeostatic conditions, Treg cells primarily prevent inflammatory responses by suppressing the activity of effector T cells (45). However, in an inflammatory environment, the phenotype of intestinal Treg cells can be modulated by inflammatory cytokines (46). For instance, the inducible co-stimulatory molecule (ICOS) stabilizes intestinal Treg cells through a CNS2-dependent mechanism (44). Additionally, Treg cells expressing CD39 and CD73 convert extracellular ATP into the immunosuppressive adenosine, with CD73 expression induced by TGF-β, thereby enhancing Treg cell-mediated immunosuppression (47). Studies have demonstrated that mice lacking cytotoxic T-lymphocyte-associated protein 4 (CTLA-4), IL-35, IL-10, or LAG-3 fail to effectively suppress T cell proliferation *in vitro* and cannot prevent chronic T cell-mediated colitis (48). Moreover, the loss of immune regulatory mechanisms in specific regions of Treg cells can lead to excessive production of pro-inflammatory cytokines, further driving chronic inflammation (49). For example, the specific deletion of CTLA-4 in Foxp3+ Treg cells results in lymphoproliferative disorders and multi-organ autoimmunity, while the absence of IL-10 in Foxp3+ Treg cells induces microbiota-driven colitis (50).

**Figure 2:**
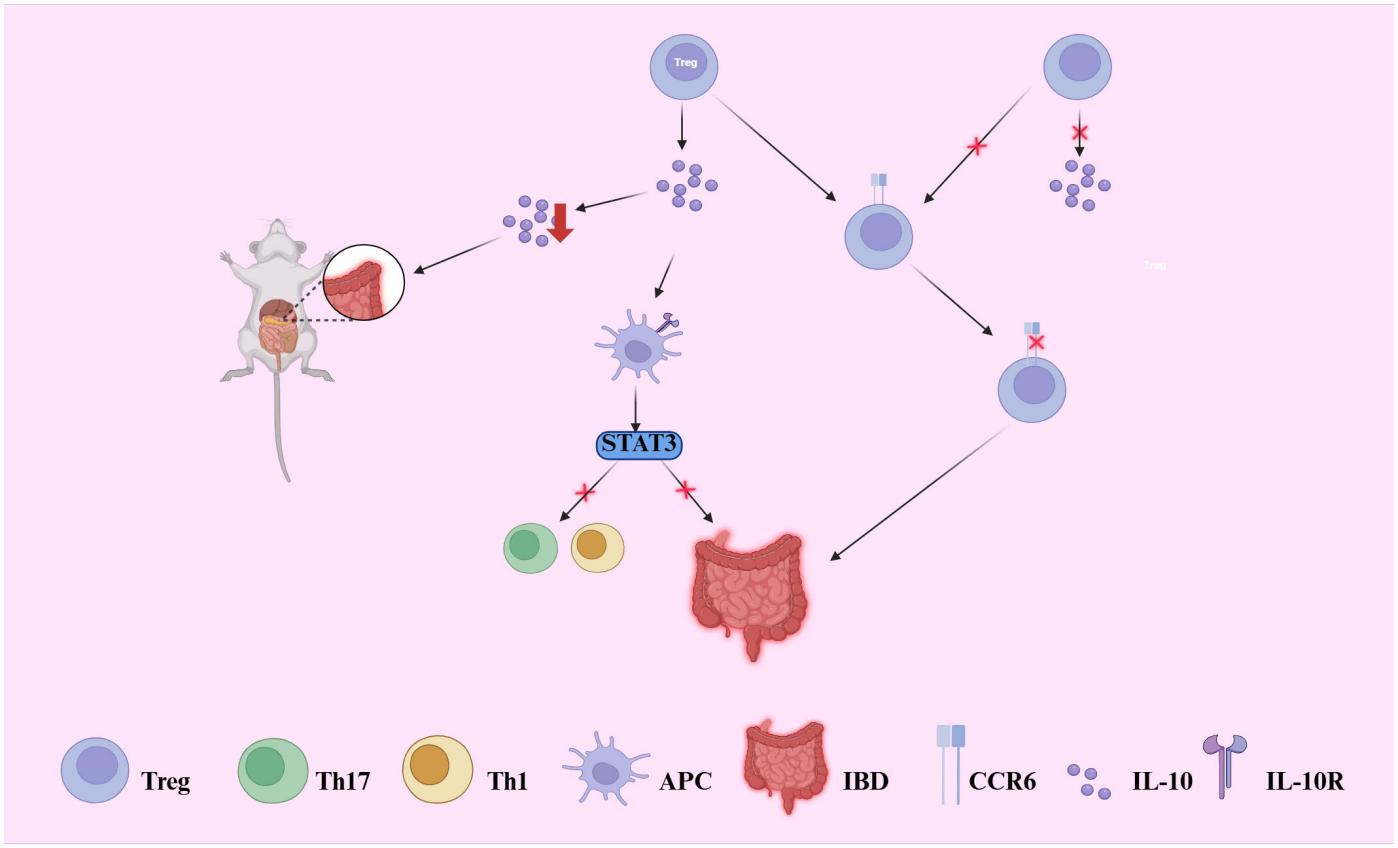
Illustrates how regulatory T (Treg) cells maintain intestinal immune tolerance through IL-10 and TGF-β secretion. IL-10 binds to IL-10 receptors (IL-10R) on antigen-presenting cells (APCs), activating the STAT3 pathway and suppressing Th1/Th17-driven inflammation. Disruption of this axis leads to uncontrolled immune activation. IL-10 deficiency in mice induces spontaneous colitis, and IL-10 signaling defects are linked to IBD in humans. Moreover, the chemokine receptor CCR6, predominantly expressed on IL-10-producing Treg cells, is critical for their homing to the gut. Loss of CCR6 reduces local Treg accumulation and IL-10 production, thereby exacerbating intestinal inflammation.

Treg cells maintain immune tolerance by secreting anti-inflammatory cytokines, such as IL-10 and TGF-β, which suppress excessive immune responses (51). IL-10, the primary immunosuppressive cytokine secreted by intestinal Treg cells, binds to the IL-10 receptor (IL-10R) on antigen-presenting cells (APCs) (52). This interaction activates the STAT3-dependent signaling pathway, inhibiting Th1, Th17, and Th1/Th17-mediated inflammatory responses, thereby limiting the activation of inflammatory cells (37). Studies have demonstrated that IL-10 deficiency leads to the development of intestinal inflammation in mice, and defects or dysfunction in the IL-10 pathway are closely associated with disease progression in human IBD patients (53). Consequently, IL-10 plays a central role in maintaining immune tolerance and significantly contributes to the immunoregulatory landscape in the pathogenesis of IBD (see **Figure 2**) (52). The immunosuppressive function of Treg cells is further modulated by various co-stimulatory receptors, including the common co-stimulatory receptors PD-1 and CTLA-4 found on the surface of Treg cells (54). The use of CTLA-4 inhibitors in cancer immunotherapy is known to induce colitis as a side effect, whereas blocking the PD-1 pathway does not demonstrate a significant association with the development of colitis (54). Notably, PD-1 primarily exerts its effects in IBD by regulating IL-10-secreting Tr1 cells (51).

#### 2.3.2 Dysregulation of Treg cells in inflammatory bowel disease

Dysfunction of regulatory T (Treg) cells can lead to uncontrolled immune responses, thereby triggering the onset and progression of inflammatory bowel disease (IBD) (53). Numerous studies have demonstrated that defects in Treg cell function are closely associated with IBD, particularly mutations in the regulatory factor IL-10 and the signaling pathway involving Foxp3 (55). IL-10 plays a crucial role in regulating Treg cell function; Treg cells lacking the IL-10 receptor are more susceptible to developing colitis (53, 55). Furthermore, when Treg cell depletion or TGF-β1 signaling is impaired, symptoms of colitis are exacerbated, underscoring the critical role of Treg cells in suppressing intestinal inflammation. During episodes of inflammation in IBD, the number of Treg cells typically increases, reflecting the body’s self-regulatory response to inflammation (56). However, despite the increase in Treg cell numbers, their functionality may be compromised due to alterations in the inflammatory microenvironment (56). For instance, Treg cells deficient in CCR6 exhibit impaired homing to the gut, leading to a decrease in their suppressive function and exacerbating the progression of colitis (57). CCR6 is predominantly expressed on IL-10-producing Treg cells, and its deficiency may result in a localized decrease in IL-10 levels, further weakening the immune regulatory function of Treg cells (see **Figure 2**) (57).

In addition to IL-10, Foxp3 serves as a critical regulatory factor for the function of Treg cells (58). The dysfunction or reduced prevalence of Foxp3+ Treg cells is closely associated with the development of inflammatory bowel disease (IBD) (59). Studies utilizing mouse models have demonstrated that the transplantation of Foxp3+ Treg cells can effectively suppress colitis induced by CD4+ CD45RBhi T cells (53). This finding underscores the significant role of Foxp3+ Treg cells in maintaining immune homeostasis and inhibiting intestinal inflammation (60). During the progression of colitis, CD4+ CD45RBlo T cells (Treg cells) exert their anti-inflammatory effects through the secretion of TGF-β and IL-10 (61). These investigations highlight the strong correlation between the imbalance of Treg cell subsets and the advancement of IBD (62).

The suppressive function of Treg cells in IBD, along with their dysfunction in immune overreaction, opens new avenues for therapeutic interventions (59). Strategies aimed at restoring Treg cell function or enhancing their suppressive activity, particularly through the modulation of the IL-10 and Foxp3 signaling pathways, may represent promising approaches for future IBD therapies (63).

### 2.4 Balance of Th17 cells with Treg cells

Recent studies have demonstrated that CCR7 is pivotal in regulating the balance between Th17 and Treg cells in Crohn’s disease mouse models exhibiting ileitis (64). Dysfunction of CCR7 can disrupt this balance, exacerbating intestinal inflammation (65). The lineage-determining transcription factors of Th17 and Treg cells, specifically RORγt and Foxp3, are critical for maintaining the Th17/Treg equilibrium (53). Research indicates that Foxp3+ Treg cells can express the retinoic acid receptor-related orphan receptor RORγt, which enables their potential differentiation into Th17 cells (53). In a transfer colitis mouse model, Treg cells, aided by TGF-β, diminish the reactivity of Th17 cells, thereby inhibiting the onset of intestinal inflammation (66). RORγt serves as a signature transcription factor for Th17 cells, while Foxp3 is a specific marker for Treg cells (67). The stability of RORγt and Foxp3 is modulated by various post-translational modifications, including ubiquitination, acetylation, and phosphorylation, which play a crucial role in sustaining the Th17/Treg balance (66, 67).

Recent studies have demonstrated that TAZ and TEAD1, both members of the TEAD transcription factor family, significantly influence the reciprocal differentiation of Th17 and Treg cells (68). In Th17 cells, TAZ serves as a cofactor for RORγt, promoting the instability of Foxp3 (69). Conversely, in Treg cells, TEAD1 maintains Foxp3 stability by sequestering TAZ, thus regulating the Th17/Treg balance (70). Furthermore, other transcription factors, including BACH2, YY1, and Batf3, also play critical roles in modulating this balance (71). BACH2 facilitates Treg cell differentiation while inhibiting effector T cell differentiation; notably, BACH2-deficient mice exhibit severe inflammation (72). YY1 diminishes Treg cell function by inhibiting Foxp3 transcriptional activity, thereby impacting immune regulation (73). Additionally, Batf3 enhances Th17 cell differentiation by suppressing Foxp3 transcription, further amplifying immune responses (74).

In conclusion, the imbalance between Th17 and Treg cells in autoimmune diseases, such as inflammatory bowel disease (IBD), is a critical factor contributing to immune overreaction and chronic inflammation (75). Modulating the balance between these cells, particularly through the regulation of RORγt, Foxp3, and other related transcription factors, may offer novel strategies and targeted intervention points for the treatment of IBD (76).

## 3 Gut microbiota and immunomodulation

### 3.1 Composition and function of the gut microbiota

#### 3.1.1 Differences between normal gut microbiota and IBD patients

Dysbiosis of the gut microbiota is recognized as a pivotal factor in the initiation of inflammation within the pathogenesis of inflammatory bowel disease (IBD) (77). The gut microbiota comprises trillions of microorganisms, predominantly anaerobic bacteria, which outnumber facultative anaerobes and aerobic bacteria by approximately 100-fold (78). Although over 50 bacterial phyla have been identified in the human gut, two phyla predominantly shape the gut microbiome: *Bacteroidetes and Firmicutes* (79). The relative proportions of these bacterial groups remain relatively stable in healthy individuals; however, significant alterations in the composition of the gut microbiota occur in IBD patients (79). Notably, the abundance and diversity of *Firmicutes* are frequently disrupted in IBD patients (77). Several studies report decreased diversity and loss of butyrate-producing *Firmicutes* in active IBD, though stool vs mucosal profiles differ (80). Many bacteria within the *Firmicutes phylum* are crucial producers of short-chain fatty acids (SCFAs), such as butyrate and acetate, which are extensively studied for their potent anti-inflammatory properties (78). SCFAs not only supply energy to intestinal epithelial cells but also suppress inflammation by modulating immune responses, thereby maintaining intestinal immune homeostasis (81). The observed reduction in *Firmicutes* implies a potential decline in SCFA synthesis, which may represent a critical mechanism contributing to the exacerbation of intestinal inflammation in IBD patients (78). A study conducted in 2019 revealed that the abundance and diversity of *Bacteroidetes* were often lower in IBD patients, a change that was significantly more pronounced compared to the proportion of *Bacteroides* species typically found in healthy individuals (79). This alteration may lead to a decrease in intestinal immune tolerance, further precipitating immune system attacks on the gut (78).

Importantly, within the *Firmicutes phylum*, Indigenous *Clostridium clusters IV and XIVa (Firmicutes)* are central regulators of mucosal immunity, inducing colonic Tregs via epithelial TGF-β and dendritic cell cooperation. Their depletion—especially of *Faecalibacterium prausnitzii*—in IBD patients impairs Treg induction and compromises intestinal tolerance, exacerbating inflammation (82).

Recent studies have increasingly focused on the mechanisms by which the microbiota regulates the balance between Th17 and Treg cells (75, 83). Dysbiosis in the gut of patients with inflammatory bowel disease (IBD), particularly the reduction in short-chain fatty acids (SCFAs), may disrupt intestinal immune balance by impairing Treg cell function or activating Th17 cells, thereby exacerbating disease progression (77, 78). This discovery opens a new avenue for IBD treatment: restoring the balance of the gut microbiota and promoting SCFA synthesis may enhance Treg cell function and inhibit the excessive activation of Th17 cells. Nevertheless, microbial shifts are context-dependent, influenced by disease subtype, disease activity, and sampling site, highlighting the need for cautious interpretation of phylum-level changes such as *Firmicutes/Bacteroidetes* ratios.

#### 3.1.2 The relationship between pathogenic bacteria and the occurrence of IBD

In immunodeficient mice, the intestinal epithelial barrier is compromised, allowing the gut microbiota to penetrate the lamina propria and subsequently drive inflammation (84). The transfer of CD4+ CD45RBhi T cells into immunodeficient mice with a reduced gut microbiota induces less severe colitis, whereas it does not induce colitis in germ-free mice (85). This observation suggests that the gut microbiota plays a crucial role in the induction of colitis (86). Among the gut microbiota, *Enterobacteriaceae*, particularly certain adherent-invasive *Escherichia coli* (AIEC) strains, are associated with the ileal mucosa in patients with Crohn’s disease (87). These bacteria are considered potential pathogens due to their ability to replicate within epithelial cells *in vitro*. Furthermore, *Mycobacterium avium subspecies paratuberculosis* has been investigated as a potential cause of Crohn’s disease, as it can induce chronic granulomatous enteritis in sheep and cattle (88). Similarly, the isolation of a highly invasive strain from patients has implicated *Fusobacterium nucleate* in its specific association with ulcerative colitis and the development of colorectal cancer (89). Nevertheless, a clear causal relationship between these pathogens and inflammatory bowel disease (IBD) remains to be established (90).

### 3.2 Mechanisms of intestinal dysbiosis

Numerous studies have demonstrated that gut microbiota plays a crucial role in the remission and progression of inflammatory bowel disease (IBD) (91), while inflammation induced by IBD, in turn, drives the loss of microbial diversity (92). Culture-independent analyses of the IBD microbiome have consistently revealed features associated with the disease, including an increased ratio of *Firmicutes* to *Bacteroidetes* when compared to healthy individuals (93). In germ-free mice, the strongest induction of cell populations, including RORγt+ FoxP3- Th17 cells and FoxP3+ Treg cells, was observed upon colonization with human IBD-associated microbiota, which is rich in Th17 cells (94). Microbiota that strongly induce Th17 cells exacerbate colitis in mouse models (95). Most intestinal Th17 cells are specific to microbial antigens, and colonization of germ-free mice also increases the frequency of intestinal FoxP3+ Treg cells (96). Specialized subsets of Treg cells in the lamina propria are distinguished by the expression of different transcription factors (97). RORγt+ Treg cells, which are microbiota-dependent, are abundant in intestinal tissues and exhibit strong suppressive and stable phenotypes (91). Mice with selective defects in RORγt in Treg cells demonstrate that RORγt+ Treg cells are essential for maintaining tolerance to the microbiota, and microbiota that promote the induction of RORγt+ Treg cells can protect mice from colitis (93). In IBD mouse models, the composition of IBD-associated microbiota can induce Th17-biased effector T cell responses and exacerbate disease severity. Furthermore, complete human fecal microbiota from both healthy donors and IBD patients can induce intestinal inflammation in susceptible mice (98).

### 3.3 Molecular mechanisms of microbial modulation on the Th17/Treg axis

Studies have demonstrated that in the presence of Toll-like receptor 9 (TLR9), gut microbiota-derived DNA can directly induce and promote the differentiation of Th17 cells, inhibit regulatory T (Treg) cells, and exacerbate intestinal inflammation (99). Additionally, certain gut microbiota has been found to alter the Th17/Treg cell balance by releasing polysaccharide A (PSA), shifting the balance toward Treg cells. *Bacteroides fragilis* employs its zwitterionic polysaccharide A (PSA) as a key symbiosis factor to maintain immune homeostasis. Processed and presented via MHC II by antigen-presenting cells, PSA activates CD4+ T cells and drives their differentiation into anti-inflammatory Foxp3+ regulatory T (Treg) cells and interleukin-10 (IL-10) production, as established by Mazmanian et al. This mechanism rectifies immunologic deficits in germ-free mice, promotes immune balance, and confers protection in experimental colitis models (100). These findings, together with the established role of *B. fragilis* PSA, underscore a remarkable convergence wherein phylogenetically diverse commensal bacteria have evolved distinct molecular strategies to actively promote the development and expansion of regulatory T cells. This collective function is crucial for enforcing intestinal immune tolerance and maintaining systemic immune homeostasis (101).

Seminal work by Atarashi et al. demonstrated that spore-forming *Clostridia clusters IV and XIVa* expand colonic Foxp3+ Tregs and protect against experimental colitis by stimulating epithelial-derived TGF-β and facilitating dendritic cell–mediated differentiation of IL-10–producing Tregs (82). Additionally, *Clostridia* can suppress the release of the pro-inflammatory cytokine IL-17 via short-chain fatty acids (SCFAs), thereby reducing Th1 and Th17 cell differentiation (102). The short-chain fatty acid butyrate, a major microbial metabolite, potently induces the *de novo* differentiation of colonic regulatory T (Treg) cells by acting as a histone deacetylase (HDAC) inhibitor. As definitively shown by Furusawa et al., this HDAC inhibition causes histone hyperacetylation at the Foxp3 locus, which epigenetically enhances its transcription and stabilizes Treg cell lineage commitment (103). In parallel, SCFAs can augment the synthesis of TGF-β1 in intestinal epithelial cells, which provides a complementary signaling axis to further promote the development and function of Treg cells (104). Short-chain fatty acids (SCFAs) can influence ATP levels through G protein-coupled receptors (GPCRs), such as GPR43, or via the mechanistic target of rapamycin (mTOR), thereby enhancing Treg cell differentiation. The use of specific SCFAs, either alone or in combination, has been shown to prevent experimental colitis in mice, which is associated with a reduction in pro-inflammatory cytokine production and the induction of Foxp3+ Treg cells in the colon (105). Therefore, SCFA administration may help suppress intestinal inflammation by expanding endogenous Foxp3+ Treg cell compartments, offering a potential therapeutic approach for patients with inflammatory bowel disease (IBD).

**Figure 3:**
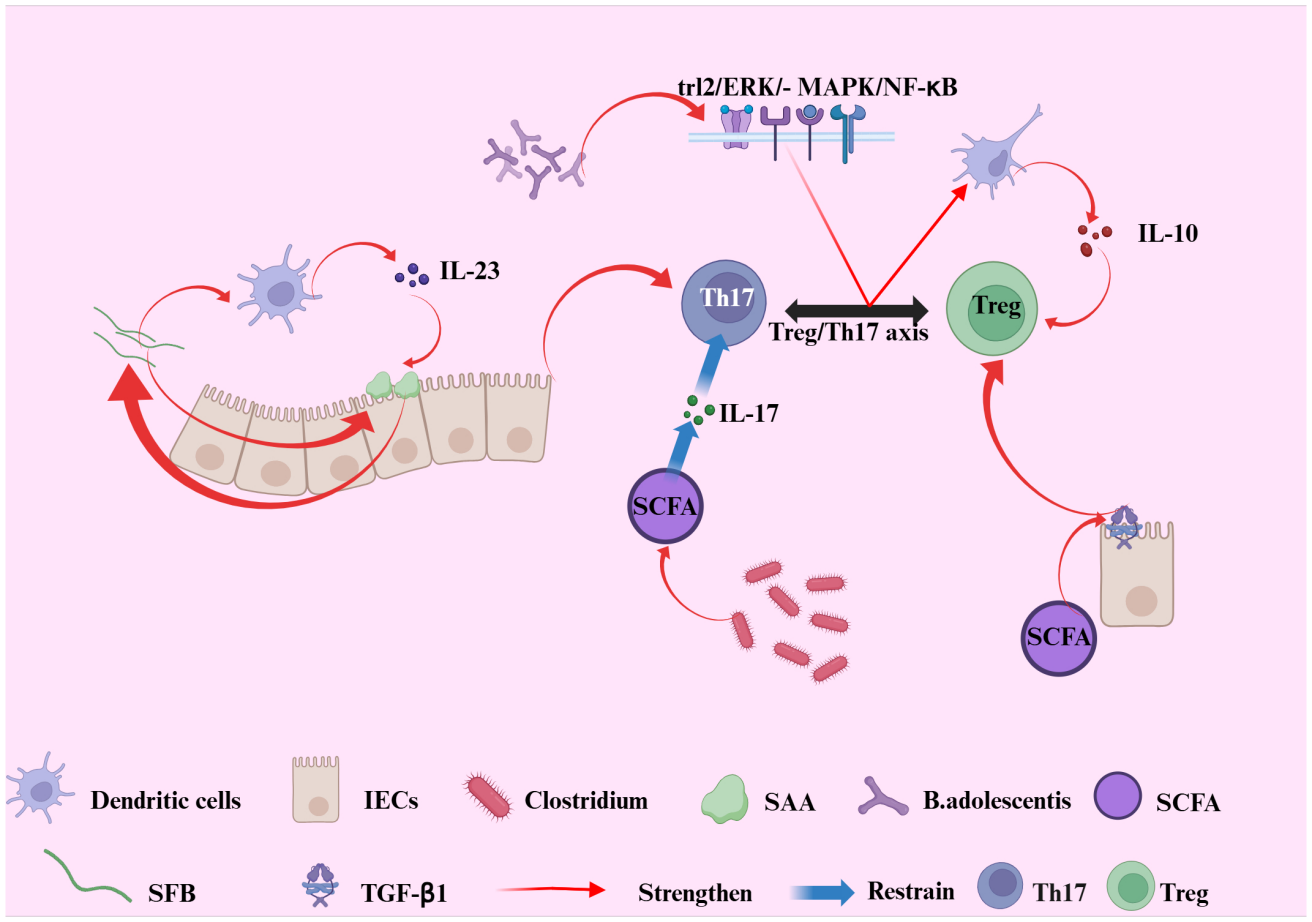
Illustrates the interplay between gut microbiota-derived signals and the Treg/Th17 axis in the context of intestinal immune regulation. Bifidobacterium adolescents transmit probiotic-mediated immune modulation to the Treg/Th17 axis via the TLR2/ERK/MAPK/NF-dB signaling cascade, inducing immunosuppressive polarization of macrophages and enhancing IL-10 production. Segmented filamentous bacteria (SFB) promote Th17 cell differentiation through intestinal epithelial cell (IEC)-derived serum amyloid A (SAA) and dendritic cells (DCs). In turn, IL-23 secreted by DCs amplifies SAA production and IL-17 secretion, reinforcing Th17 differentiation and sustaining mucosal inflammation. In parallel, SCFAs can augment the synthesis of TGF-β1 in intestinal epithelial cells, which provides a complementary signaling axis to further promote the development and function of Treg cells. Conversely, commensal Clostridium species inhibit the release of pro-inflammatory IL-17 via the production of short-chain fatty acids (SCFAs), thereby suppressing Th1 and Th17 cell differentiation. Collectively, these interactions highlight the crucial role of gut microbial signals in maintaining intestinal immune homeostasis through dynamic regulation of the Treg/Th17 axis.

Probiotics can suppress IL-17 production and function by reducing IL-23 secretion, thereby improving intestinal inflammation through this mechanism (106). Research has demonstrated that *Bifidobacterium adolescents* can mediate probiotic-induced adaptive immune regulation of the Treg/Th17 axis via the TLR2/ERK/MAPK/NF-κB signaling pathway, which stimulates the immune-suppressive polarization of macrophages and the secretion of the cytokine IL-10 (107). Furthermore, probiotics may offer an alternative method to promote the induction of Foxp3+ Treg cells in patients with inflammatory bowel disease (IBD) (108). In a study conducted by Kwon et al., a probiotic mixture comprising *Lactobacillus acidophilus*, *Lactobacillus casei*, *Lactobacillus reutter*, *Bifidobacterium adolescents*, and *Streptococcus thermophilus* significantly increased the number of tolerogenic dendritic cells (DCs) and Treg cells in mesenteric lymph nodes, while concurrently reducing the expression of pro-inflammatory cytokines and the proliferation of CD4+ T cells (see **Figure 3**) (109).

The gut microbiota has emerged as a novel therapeutic target for inflammatory bowel disease (IBD) due to its influence on immune system function, particularly in the regulation of T helper 17 (Th17) and regulatory T (Treg) cell differentiation and function (110). Dysbiosis of the gut microbiota disrupts the Th17/Treg balance via multiple mechanisms, thereby facilitating the onset and progression of IBD (75). Further research is essential to deepen our understanding of these mechanisms, providing new theoretical foundations and methodologies for IBD treatment. Moreover, the gut microbiota, through its metabolites such as short-chain fatty acids (SCFAs) and specific bacterial species, regulates the balance between Th17 and Treg cells, thereby modulating immune and inflammatory responses (105). SCFAs influence the immune system through various pathways, particularly by promoting Treg cell differentiation and function, which alleviates intestinal inflammation (111). *Segmented Filamentous Bacteria* (SFB) promote Th17 cell differentiation through intestinal epithelial cell (IEC)-derived serum amyloid A (SAA) and dendritic cells (DCs), reinforcing mucosal inflammation (112). Furthermore, the roles of intestinal microbes such as *Clostridia* and SFB highlight the significant impact of dysbiosis on immune balance (113). In the treatment of IBD, modulation of the gut microbiota, especially through the supplementation of probiotics or SCFAs, may represent a promising strategy to restore immune tolerance and improve clinical outcomes (114). While these molecular mechanisms elucidate the how, the following section synthesizes the key supporting evidence for these pathways (**Table 1**) and explores their translational potential into microbiota-targeting therapies (**Table 2**).

**Table 1:** Key mechanistic pathways in gut microbiota-mediated immunomodulation of the Treg/Th17 axis.

Regulatory mechanism	Regulatory flora	Molecular mediators	Immune bias	Human/animal evidence	Key references
GPR43/GPR109A	SCFA-producing bacteria (*Clostridia, F. parasitize*)	Butyrate, acetate (GPCR signaling)	↑Treg	Mouse DSS colitis; IBD patient biopsies	(144)
VEGF/Nrp-1	*Commensal flora*	VEGF-Nrp1 signaling supports immune tolerance	↑Treg	Mouse gut mucosa studies	(145)
IL-10/STAT3	*Bifidobacterium breve*	IL-10 secretion activates STAT3, reduces inflammation	↑Treg	Mouse models of colitis; human PBMCs	(146)
Foxp3	*Clostridia clusters IV & XIVa*	Butyrate/SCFAs enhance Foxp3 stability	↑Treg	GF mice colonization; IBD patients	(82)
PD-1/PD-L1	*Lactobacillus casei*	PD-1/PD-L1 signaling adjusts Treg function	↑Treg	Mouse tumor/colitis models	(147)
SCFA/HDAC	*Rosebury* spp.*, Butyric coccus*	Butyrate inhibits HDAC → ↑Foxp3	↑Treg	IBD biopsies; mouse colitis	(148)
Indole/Ahr	*Bacteroides* spp.	Indole metabolites act on Ahr → Treg induction	↑Treg	Mouse colitis; Ahr ligands	(149)
Ahr	*Lactobacillus reutter*	Tryptophan metabolites activate Ahr	↑Treg	Mouse colitis & Treg expansion	(150)
Want/β-catenin	*Lactobacillus plantarum*	Regulates epithelial integrity, supports Tregs	↑Treg	Mouse gut barrier & Treg data	(151)
CD39/CD73	*Clostridium cluster IV*	Adenosine generation inhibits inflammation	↑Treg	Human *in vitro*; mouse colitis	(146)
Retinoic Acid	*Clostridium* spp.	RA enhances tolerance state	↑Treg	Mouse colitis; DC-Treg coculture	(152)
AMPK	*F. parasitize*	AMPK inhibits mTOR → ↑Treg	↑Treg	IBD stool samples; mouse models	(147)
OX40/OX40L	*Pathogenic Proteobacteria*	OX40L signaling suppresses Tregs	↓Treg	IBD mucosa samples	(151)
STAT5	*Clostridium butyric*	Butyrate enhances STAT5 → ↑Foxp3	↑Treg	Mouse probiotics studies	(145)
TLR9/MyD88	*Bifidobacterium infants*	CpG DNA → Treg induction	↑Treg	Mouse DSS colitis	(153)
TGF-β/Smad	*Clostridium clusters IV & XIVa*	TGF-β cooperates with DCs to induce Tregs	↑Treg	Mouse (GF colonization); Human (commensal studies)	(82)
BATF/IRF4	*Lactobacillus rhamnoses*	BATF/IRF4 TF promote Th17 differentiation	↑Th17	Mouse models	(154)
TNF-α/NF-κB	*Pathogenic Bacteroides*	NF-κB activation → pro-inflammatory Th17 response	↑Th17	Mouse colitis; IBD mucosa	(144)
IL-23/IL-23R	SFB, *Enterobacteriaceae*	IL-23 maintains Th17 cells	↑Th17	EAE/colitis mouse models	(152)
CCR6/CCL20	*B. vulgates*	Chemokine axis guides Th17 migration	↑Th17	Mouse mucosal migration studies	(151)
mTORC1	*A. municipia*	Promotes Th17 proliferation	↑Th17	Mouse IBD and metabolic models	(155)
TGF-β + IL-6	SFB	Cytokine synergy → Th17 differentiation	↑Th17	GF mice colonized with SFB	(156)
PGE2/EP4	*Fusobacterium nucleate*	PGE2 amplifies Th17 inflammation	↑Th17	Human CRC/IBD samples	(145)
IL-6/STAT3	SFB	IL-6 + IL-12 → Th17 differentiation	↑Th17	Mouse SFB colonization	(153)
TLR2/TLR4	*E. coli, B. fragili*s	LPS, PSA regulate Th17/Treg	↑Th17(pathogens)/↑Treg (commensals)	Mouse colitis, human stool	(153)
SAA/SFB	SFB	SAA-induced epithelial Th17 activation	↑Th17	Mouse intestinal epithelial cells	(152)
LPS-TLR4/IL-1β	*Pathogenic E. coli*	LPS, IL-1β induce Th17 bias	↑Th17	Mouse colitis, IBD patients	(144)
NF-κB	*Candida albicans*	β-glucans activate NF-κB → Th17 amplification	↑Th17	Mouse colitis; microbiota shifts in IBD	(144)

This table catalogues molecular mechanisms through which commensal and pathogenic gut microbiota influence intestinal immune homeostasis in inflammatory bowel disease (IBD) by modulating the balance between regulatory T (Treg) and T helper 17 (Th17) cells. The summarized pathways mediate host-microbe communication through: (i) microbial metabolites (e.g., SCFAs, tryptophan derivatives); (ii) pattern recognition receptors (e.g., TLR2, TLR4, TLR9); (iii) epigenetic mechanisms (e.g., HDAC inhibition); and (iv) canonical immune signaling axes (e.g., TGF-β/Smad, IL-6/STAT3, IL-23/Rot), which collectively regulate CD4^+^ T cell differentiation.

The table illustrates how certain commensal bacteria (e.g., Clostridium spp., Faecalibacterium prausnitzii, Bifidobacterium spp.) activate tolerogenic pathways (e.g., Ahr, GPR43, STAT5) that promote Foxp3^+^ Treg cell induction, while other species (e.g., Segmented Filamentous Bacteria, Fusobacterium nucleate) activate pro-inflammatory pathways (e.g., IL-23/STAT3) that drive Th17 responses. Evidence from both experimental models and human studies indicates that these mechanisms contribute to epithelial barrier integrity, inflammatory cascades, and disease pathogenesis in IBD, providing a mechanistic foundation for microbiota-targeted therapeutic interventions.

**Table 2:** Clinical evidence for microbiota-targeted interventions and their impact on Th17/Treg balance in inflammatory bowel disease.

Intervention	Study design	Population (CD/UC)	Sample size (N)	Primary outcome	Effect on Th17/Treg biomarkers	Safety
Bifidobacterium infants	8-week randomized controlled trial (RCT)	UC	77	Clinical remission and endoscopic improvement	↑ Tregs (Foxp3+); ↓ Th17 cytokines (IL-17A)	Well-tolerated; no related serious adverse events (SAEs)
Clostridium butyric	Prospective cohort	CD & UC	50	Improvement in clinical activity index	↑ Treg frequency; ↑ IL-10; ↓ IL-6, IL-17	Safe; mild gastrointestinal discomfort (15% of patients)
Fecal microbiota transplantation (FMT)	Open-label trial	UC	60	Clinical and endoscopic remission	↑Treg-associated SCFA levels; ↓ Th17-related cytokines	Generally safe; transient abdominal symptoms
High-fiber diet	Prospective interventional study	CD	35	Improved symptom score; reduced C-reactive protein (CRP)	↑ SCFA production; ↑ Tregs; ↓ Th17 polarization	Safe; high adherence rate
Specific Carbohydrate Diet (SCD)	Pilot study	CD	20	Symptom improvement; reduced inflammatory markers	Microbiota shift → ↑ Treg-inducing Clostridia; ↓ Th17 response	Safe; requires dietary counseling

This table summarizes key clinical studies evaluating interventions designed to modulate the gut microbiota for the treatment of Crohn’s disease (CD) and ulcerative colitis (UC). It highlights the effects of probiotics, fecal microbiota transplantation (FMT), and dietary modifications on both clinical endpoints (e.g., clinical remission, endoscopic improvement) and immunologic biomarkers specific to the Th17/Treg axis. A consistent immunomodulatory pattern emerges across diverse interventions, demonstrating their capacity to rebalance the intestinal immune milieu towards tolerance by upregulating Treg cells and their associated anti-inflammatory mediators (e.g., IL-10, SCFAs) while concurrently suppressing pro-inflammatory Th17 responses and related cytokines (e.g., IL-17A, IL-6). The generally favorable safety profiles support the therapeutic potential of these approaches; however, heterogeneity in study designs and outcomes highlights the need for larger, randomized controlled trials to definitively establish efficacy and define optimal treatment protocols.

### 3.4 Evidence synthesis and translational prospects of microbial immunomodulation in IBD

Building upon the molecular mechanisms outlined above, the evidence for microbial immunomodulation is robustly supported by both mechanistic studies (summarized in **Table 1**) and clinical interventions (detailed in **Table 2**). The balance of the Th17 and Treg cell axes is critically regulated by the intestinal microbiota through a complex network of metabolic pathways, cytokine signals, transcription factors, and signal transduction pathways (Summarized in **Table 1**). Specific commensals, such as *Clostridium clusters*, *Bifidobacterium*, and *Faecalibacterium prausnitzii*, promote tolerogenic responses by activating specific mechanisms including the aryl hydrocarbon receptor (AhR), short-chain fatty acid (SCFA) receptors (e.g., GPR43), and STAT5 signaling pathways, leading to the induction and functional enhancement of Foxp3+ Treg cells. Conversely, pathobionts like *Segmented Filamentous Bacteria* (SFB) and *Fusobacterium* nucleated drive pro-inflammatory Th17 differentiation primarily via the IL-23/STAT3 axis, often in conjunction with TGF-β and IL-6 signaling. This mechanistic interplay is not only fundamental to mucosal immune homeostasis but also provides a rationale for microbiota-targeting therapies. As evidenced by the clinical data compiled in **Table 2**, interventions such as specific probiotics, fecal microbiota transplantation (FMT), and dietary modifications can promote a favorable shift in this immunologic balance in patients with IBD, as evidenced by increased Treg responses and suppression of Th17-related inflammation (detailed in **Table 2**). Collectively, this triad of microbial mechanisms, immunological effects, and clinical outcomes constitutes a robust theoretical and translational framework for understanding IBD immunopathogenesis and provides a concrete foundation for developing targeted microbial or pathway-specific immunotherapeutic strategies.

## 4 Gut microbiota regulation as a therapeutic and prognostic strategy for IBD

### 4.1 The application of probiotics, a certain intestinal flora

Several reports have indicated that specific probiotic strains may enhance the management of inflammatory bowel disease (IBD). One clinical trial identified *Escherichia coli* (*E. coli*) as a potentially effective probiotic for ulcerative colitis (UC). In a double-blind, placebo-controlled study, UC patients in remission received bacterial preparations containing live *E. coli* or mesalamine tablets for a duration of five days. Following a 12-month follow-up, no significant differences in UC relapse rates were observed between the *E. coli* and mesalamine treatment groups. Consequently, *E. coli* may serve as a novel probiotic formulation, offering an alternative to mesalamine (115).

Lactic acid bacteria species, such as *Lactobacillus rhamnoses* (*GG*), have been utilized in the treatment of inflammatory bowel disease (IBD) (116). Oral administration of *L. rhamnoses* (*GG*) has been demonstrated to reduce levels of inflammatory cytokines and improve colonic histological scores, thereby preventing the recurrence of colitis in rats undergoing antibiotic treatment (117). Furthermore, a study involving a colitis mouse model indicated that *Enterococcus Ludwig* (*E. Ludwig*) effectively alleviates symptoms of colitis (118). In a dextran sulfate sodium (DSS)-induced colitis mouse model, metronidazole emerged as the most effective treatment for reducing colitis compared to other antibiotics, which was associated with an increase in the diversity of gut microbiota species (119). The administration of E. Ludwig promotes Treg cell differentiation through its metabolites and enhances immune tolerance, consequently reducing susceptibility to DSS-induced colitis in mice. Conversely, *Candida albicans* (*C. albicans*), typically a beneficial species within the human gut microbiota, can become pathogenic in immunocompromised hosts. The hyphae of *C. albicans* directly activate B cells to express IgG1 and IL-6, leading to the differentiation of Th17 cells and ultimately facilitating bacterial clearance (120).

Probiotics, such as *Bifidobacterium longum*, have been shown to increase Treg cells, thereby inducing IL-10 production and improving the mucosal immune response in inflammatory bowel disease (IBD) (121). Probiotics, which consist of a mixture of beneficial bacteria or yeast, are utilized to restore the balance of gut microbiota and are considered advantageous for health. Nevertheless, the evidence supporting the efficacy of probiotic therapy in IBD remains inconclusive (122). The most compelling findings originate from post-operative ulcerative colitis (UC) patients who have undergone ileal pouch-anal anastomosis (IPAA). These patients are at a high risk for developing paucities, an inflammation of the ileal pouch, and probiotics have demonstrated effectiveness in preventing this complication following successful antibiotic treatment (123). However, fundamental questions regarding the optimal composition of probiotics, the timing of administration, and the persistence of therapeutic responses remain unanswered (124).

### 4.2 Dietary interventions

Vitamin D, short-chain fatty acids (SCFAs), and metabolites of the gut microbiota exhibit anti-inflammatory properties that protect the intestinal epithelium, promoting Treg cell differentiation and upregulating the expression of anti-inflammatory cytokines (105). Nutrient intake is one of the most significant factors influencing gut microbiota and intestinal mucosal immunity (125). For instance, a preliminary study on human subjects demonstrated that moderate high-salt intake increased Th17 cell levels by reducing the survival rate of *Lactobacillus* species in the gut (126). Excessive sugar intake mediates an increase in cytokines such as IL-1β and Th17 cells, thereby inducing inflammation (127). Both ketogenic and Mediterranean diets have been shown to reduce intestinal Th17 cells by altering the gut microbiome (128). SCFAs—including acetate, propionate, and butyrate—are metabolic end-products of indigestible carbohydrate and protein fermentation in the gastrointestinal tract. These metabolites promote Treg cell differentiation, suppress intestinal inflammation, and play a crucial role in maintaining intestinal immune homeostasis through anti-inflammatory and immunosuppressive functions (105).

Excessive caloric intake has been demonstrated to promote Th17 cell differentiation by enhancing glycolysis and activating the mTOR signaling pathway, thereby fostering an inflammatory environment and influencing the composition of the gut microbiota. Conversely, caloric restriction inhibits Th17 cell differentiation through the activation of the AMPK pathway, without adversely affecting Treg cell differentiation (129). Research indicates that interventions designed to increase Treg cells or modulate the Th17/Treg balance can mitigate obesity-related immune issues and inflammatory bowel disease (IBD) (51). Nutrient deficiencies result in defects in T cell differentiation, whereas high-fat diets promote Th17 cell differentiation by regulating ACC1, which upregulates the expression of IL-17A, IL-23R, and CCR6 (130). Treg cell proliferation and activation depend on exogenous fat uptake and are largely independent of cellular glycolipid metabolic pathways, remaining relatively unaffected by high-calorie dietary metabolic shifts (131). Consequently, under high-calorie conditions, CD4^+^ T cells preferentially differentiate into Th17 cells, creating a pro-inflammatory environment conducive to IBD onset and progression (132). Additionally, leptin has been reported to activate the mTOR pathway in CD4+ T cells, promoting the differentiation of Th17 and Th1 cells while inhibiting Treg cell differentiation. Galgano et al. also found that leptin influences T cell vitality and growth by regulating Bcl-2, Th1/Th17 cytokines, and the AKT-mTOR pathway (133). This effect of leptin may occur through the increased expression of Glut1 and HIF-1α, which promotes glycolysis and drives Th17 cell differentiation. Therefore, dietary control plays a crucial role in the adjunctive treatment of IBD (134).

### 4.3 Fecal microbiota transplantation

#### 4.3.1 Preclinical evidence for Treg/Th17 balance from animal models

Research involving microbiota colonization and transplantation in IBD mouse models has shown a notable increase in the proportion of RORγt+ Treg cells in the colon following transplantation, indicating that microbiota may play a crucial role in regulating intestinal immune tolerance (135). Dynamic changes in various microbiota can induce the generation of RORγt+ T regulatory (Treg) cells, whose numbers are significantly diminished in IBD models. The transplantation of over 30 different human strains of *Clostridium* into germ-free mice significantly enhanced Treg cell expansion, yielding a threefold increase (136). In contrast, the transplantation of a single *Clostridium* strain resulted in a moderate Treg response. Furthermore, the composition of the microbiota significantly influences the proportion of Th17 cells. The colonization of an IBD-associated microbiota results in an increase in Th17 cells in the gut, particularly in mice colonized with Crohn’s disease-associated microbiota, where this effect is especially pronounced (137). The rise in Th17 cells is closely linked to increased susceptibility to inflammatory diseases. Consequently, reducing the proportion of Th17 cells induced by the IBD microbiota may represent a potential therapeutic target for IBD (138). In previous studies, Rosebury intestinalis has been demonstrated to alleviate intestinal inflammation in animal models (139). Treatment of mice with inflammatory bowel disease (IBD) using *Porphyromonas gingivalis*, *Lactobacillus rhamnoses* (*GG*), *Privately* spp., or *Lactobacillus piracies* has been shown to significantly reduce intestinal inflammation, increase regulatory T (Treg) cells, and decrease T helper 17 (Th17) cells. This suggests that greater microbial diversity is essential for maintaining immune homeostasis in the host, while a reduction in biodiversity may accelerate the inflammatory processes associated with IBD (140).

#### 4.3.2 Translational nuances in immune modulation: evidence from human trials

Beyond these preclinical insights, clinical evidence from human studies reveals both the promise and complexity of modulating the gut-immune axis. Clinical and endoscopic remission in active ulcerative colitis is achievable through fecal microbiota transplantation (FMT), as evidenced by randomized controlled trials and meta-analyses. Optimal efficacy is observed with lower-gut delivery, high-dose, and multi-donor preparations. A meta-analysis confirmed FMT’s significant benefits in mild to moderate UC, though it noted a potential increase in serious adverse events that requires further validation (141). This contrasts sharply with the outcomes of targeted biologic therapies, underscoring the complexity of translating immune mechanisms into treatment. The IL-17A inhibitor eculizumab, while effective in psoriasis, has been associated with the paradoxical induction or exacerbation of Crohn’s disease in clinical reports. This is mechanistically attributed to a potential hyperactivation of Th1 pathways and a compromised mucosal barrier due to the loss of IL-17’s protective role against fungal and bacterial infections (142).Conversely, blockade of the upstream p40 subunit shared by IL-12 and IL-23 with Ustekinumab has proven effective for inducing and maintaining remission in Crohn’s disease in pivotal RCTs, highlighting that targeting a broader, more upstream pathway within the Th17 axis can be successful where direct IL-17A inhibition fails (143).This stark contrast underscores the superiority of a more nuanced, ecosystem-based approach to immune modulation, akin to the mechanisms evolved by commensal microbiota. These disparate clinical outcomes emphasize the nuanced and context-dependent nature of immune modulation.

A significant translational gap exists between the consistent efficacy of microbial interventions in animal models and their variable outcomes in human trials. The heterogeneity of FMT response and the paradoxical failure of anti-IL-17 therapy in Crohn’s disease, despite strong preclinical rationale, highlight the context-dependent complexity of human immunomodulation. This underscores the necessity for future therapies to adopt integrated, ecosystem-based strategies rather than reductionist approaches.

## 5 Conclusions and prospects

The imbalance between Th17 and Treg cells represents a fundamental immune mechanism underlying the pathogenesis of inflammatory bowel disease (IBD). Th17 cells contribute to local immune responses and intensify tissue inflammation through the secretion of pro-inflammatory cytokines such as IL-17, IL-21, and IL-22. In contrast, Treg cells play a critical role in maintaining immune tolerance by suppressing excessive immune responses. In patients with IBD, a decline in Treg cell functionality or a loss of their immunosuppressive capacity permits unchecked activation of Th17 cells, resulting in heightened immune system activation that exacerbates the pathological progression of IBD. This imbalance is particularly evident in the context of dysbiosis. The gut microbiota influences the function of both Th17 and Treg cells through direct and indirect mechanisms. Consequently, restoring the equilibrium between Th17 and Treg cells is essential for modulating the intestinal immune response and alleviating symptoms associated with IBD. The overgrowth of certain bacterial populations, such as *Firmicutes* and *Bacteroidetes*, can alter the ratio and function of Th17 and Treg cells through their metabolic products or the secretion of pathological cytokines, thereby promoting the onset of inflammatory bowel disease (IBD). Given the central role of the gut microbiota in IBD, increasing research is focusing on microbiota modulation as a novel therapeutic strategy. Probiotics, prebiotics, and fecal microbiota transplantation (FMT) have demonstrated promising therapeutic effects. FMT has been shown to restore gut microbiota balance in some IBD patients, leading to improvements in clinical symptoms. Although these treatments require further clinical validation, their potential should not be underestimated. In the future, personalized treatments will emerge as a critical direction for microbiota modulation. Due to individual variations in gut microbiota, different IBD patients may exhibit distinct immune responses and treatment outcomes. Therefore, precise modulation of the microbiota, including the selection of appropriate probiotics and therapeutic methods, will be a key task in future IBD treatment. The gut microbiota plays a crucial role in immune regulation, particularly in inflammatory bowel diseases such as colitis. Furthermore, targeted therapeutic strategies, such as phage therapy or *CRISPR/Cas* gene editing technologies, may provide more precise methods for immune modulation in IBD treatment. Combining microbiota transplantation with precision immune modulation strategies may yield more effective treatments for IBD patients.
